# Grey matter changes in medication-overuse headache before and after medication withdrawal

**DOI:** 10.1186/1129-2377-14-S1-P177

**Published:** 2013-02-21

**Authors:** F Riederer, AR Gantenbein, M Marti, R Luechinger, S Kollias, PS Sándor

**Affiliations:** 1 University Hospital Zurich, Department of Neurology, Switzerland; 2 Institute for Biomedical Engineering, Swiss Federal Institute of Technology and the University of Zurich, Switzerland; 3 Institute of Neuroradiology, University Hospital Zurich, Switzerland; 4 RehaClinic Bad Zurzach/Baden, Switzerland

## Background

Medication-overuse headache (MOH) is a complication of migraine that causes significant burden and cost. Recent studies demonstrated metabolic and structural abnormalities in MOH 1,2, including a grey matter increase in the midbrain periaqueductal grey. We hypothesised that structural changes related to MOH should return to normal after medication withdrawal in patients with significant clinical improvement (responders). In contrast, no changes were expected in patients without improvement (non-responders).

## Methods

Thirty-one MOH patients and 28 healthy controls were investigated in a longitudinal voxel-based morphometry study, comparing structural MRIs at 2 time points.

## Results

In responders, grey matter in the midbrain decreased close to normal values after withdrawal. In contrast, in non-responders no grey matter decrease from scan 1 to scan 2 was observed. At baseline, non-responders had significantly less grey matter in the right and left orbito-frontal cortex, left insula, midbrain, and the thalami compared to responders. In MOH patients grey matter in the right gyrus rectus at base line correlated positively with treatment response.

## Conclusions

Increased grey matter in the midbrain, which is involved in pain modulation, seems to decrease after successful treatment. Poor response to treatment is associated with decreased grey matter in the orbitofrontal cortex, consistent with dysfunction of this region.
